# Effect of insomnia in the major depressive disorder

**DOI:** 10.1186/s12883-022-02869-x

**Published:** 2022-09-10

**Authors:** Guohong Xu, Xiaoling Li, Caixia Xu, Guojun Xie, Jiaquan Liang

**Affiliations:** Department of Psychiatry, The Third People’s Hospital of Foshan, Foshan, Guangdong People’s Republic of China

**Keywords:** Sleep disturbance, Insomnia, Major depressive disorder

## Abstract

**Background:**

People with sleep problems are more likely to have mental disorders. This study aimed to assess the effect of insomnia on the prognosis of patients with major depressive disorder (MDD).

**Methods:**

We divided the patients into three groups according to the Insomnia Severity Index (ISI) scores. In addition, we compared the results of the Hamilton Depression Scale (HAMD) and Symptom Checklist-90 (SCL-90) scores. We evaluated the effect of insomnia at the 2nd, 4th, and 8th-week follow-up on the prognosis of MDD.

**Results:**

Fifty-five patients between 19 and 58 years old, with a diagnosis of MDD via the Structured Clinical Interview for the Diagnostic and Statistical Manual-5 (DSM-5). The ISI scores of the moderate and severe group decreased significantly (*P* < 0.05) in the 2nd week compared to the baseline. The HAMD scores in all groups improved significantly in the 2nd week.

**Conclusions:**

This study was inspired to assess insomnia as a comorbid disorder for patients with MDD, which may bring poor treatment consequences.

## Introduction

Sleep disorders afflict nearly a quarter of the global population. People with sleep problems are more likely to have mental disorders, such as bipolar disorder, generalized anxiety disorder, and especially major depressive disorder (MDD) [1]. MDD is a common mental disorder affecting more than 4% of the global population [2]. MDD is one of the most commonly diagnosed mental disorders, with a lifetime prevalence of approximately 16% [3, 4]. In many cases, sleep disorder is the main complaint of MDD [5].

Sleep disturbance is one of the most consistent symptoms associated with MDD [6]. These sleep regulation problems are not secondary to the disorders; instead, they precede depressive episodes and persist during remission. Furthermore, it has been found that depressed patients with sleep disturbance may have more severe symptoms and treatment difficulties [7]. In addition, improving sleep in patients with MDD can improve outcomes [8]. These observations suggest that it is essential for sleep medicine practitioners to realize patients with MDD and solve their sleep problems.

It is generally agreed that sleep disturbance is a symptom that can be alleviated related to the treatment of MDD. Moreover, insomnia is an important predictor of MDD recurrence and may lead to unpleasant clinical outcomes [7, 9]. It is well known that insomnia is an independent diagnostic entity, which may lead to the development of MDD [10]. Insomnia in people indicates a greater risk for MDD that persists for at least 30 years [11]. A study has confirmed the importance of insomnia as a risk factor for MDD and the necessity of early treatment of insomnia [12]. So, it is crucial to examine the role of insomnia in evaluating the prognosis of MDD.

The aim of this study was to assess the effect of insomnia on the prognosis of patients with MDD. First, we evaluated the Insomnia Severity Index (ISI), Hamilton Depression Scale (HAMD), and Symptom Checklist-90 (SCL-90) as a predictor of the effect of insomnia on MDD. ISI scale yields a total score ranging from 0 to 28. The total score is interpreted: (0–7: absence of insomnia; 8–14: slight insomnia; 15–21: moderate insomnia; and 22–28: severe insomnia [13]. The patients were divided into three groups according to the ISI scores: (8–14: slight insomnia; 15–21: moderate insomnia; 22–28: severe insomnia). In addition, we compared the results of HAMD and SCL-90 scores and evaluated the effect of insomnia at the 2nd, 4th, and 8th-week follow-up.

## Method

### Study design and participants

Participants were recruited from the department of psychiatry, the third people’s hospital in Foshan, China. Fifty-five patients between 19 and 58 years old with a diagnosis of MDD via the Structured Clinical Interview for the Diagnostic and Statistical Manual-5 (DSM-5). Patients under the age of 18 were excluded. The exclusion criteria of this study were active drug abuse, excessive use of sleep drugs, and other brain diseases, such as severe developmental disorder, Parkinson’s disease, and acquired brain injury.

The ISI is a self-report questionnaire used to assess the nature and severity of insomnia. The total score is divided into three grades: (8–14: slight insomnia; 15–21: moderate insomnia; 22–28: severe insomnia) [13]. HAMD is considered the standard measure of severity for MDD. Furthermore, many psychometric properties of the HAMD are reliable and consistently meet the established criteria. SCL-90 is widely used in mental health screening and the diagnosis of mental diseases. According to the ISI scores, we divided the MDD patients with insomnia into three groups: slight, moderate, and severe group.

### Outcomes

All patients were assessed using ISI, HAMD, and SCL-90 scores at baseline, 2nd week, 4th week, and 8th week. We used HAMD to evaluate response and remission. The remission of depressive symptoms was defined as a final HAMD score ≤ 7. A response to treatment was defined as a 50% reduction in the scale scores between the baseline and 2nd week, 4th week, and 8th-week assessments. We assessed insomnia states using ISI scores.

### Statistical analysis

For comparisons among the baseline of the slight, moderate, and severe group: age, HAMD, SCL 90, and ISI scores, one-way ANOVA was used. Categorical variables (gender, education, smoking, and educational level) were performed using χ^2^ tests. Regarding the predictive effect of insomnia on MDD recovery, we performed two-way ANOVA using the difference among baseline and 2nd week, 4th week, and 8th-week scores as ISI, HAMD, and SCL-90 scores. Bonferroni post hoc tests were performed to determine differences between groups. Frequency analysis χ^2^ was performed to assess the occurrence of remission and response. *P* < 0.05 was considered statistically significant, and the data are presented as mean ± standard deviation (SD). All data were analyzed with the SPSS Statistics, version 21.0 for Windows (IBM Corporation, Armonk, USA).

### Patient and public involvement

No patients or the public participated in the study protocol design, specific objectives or study questions, and the design or implementation of the current study. No patients or the public participated in the interpretation of the study results or the preparation of the manuscript. There are no plans to disseminate the results of the study to participants.

## Results

### Participant characteristics

**Fig. 1:**
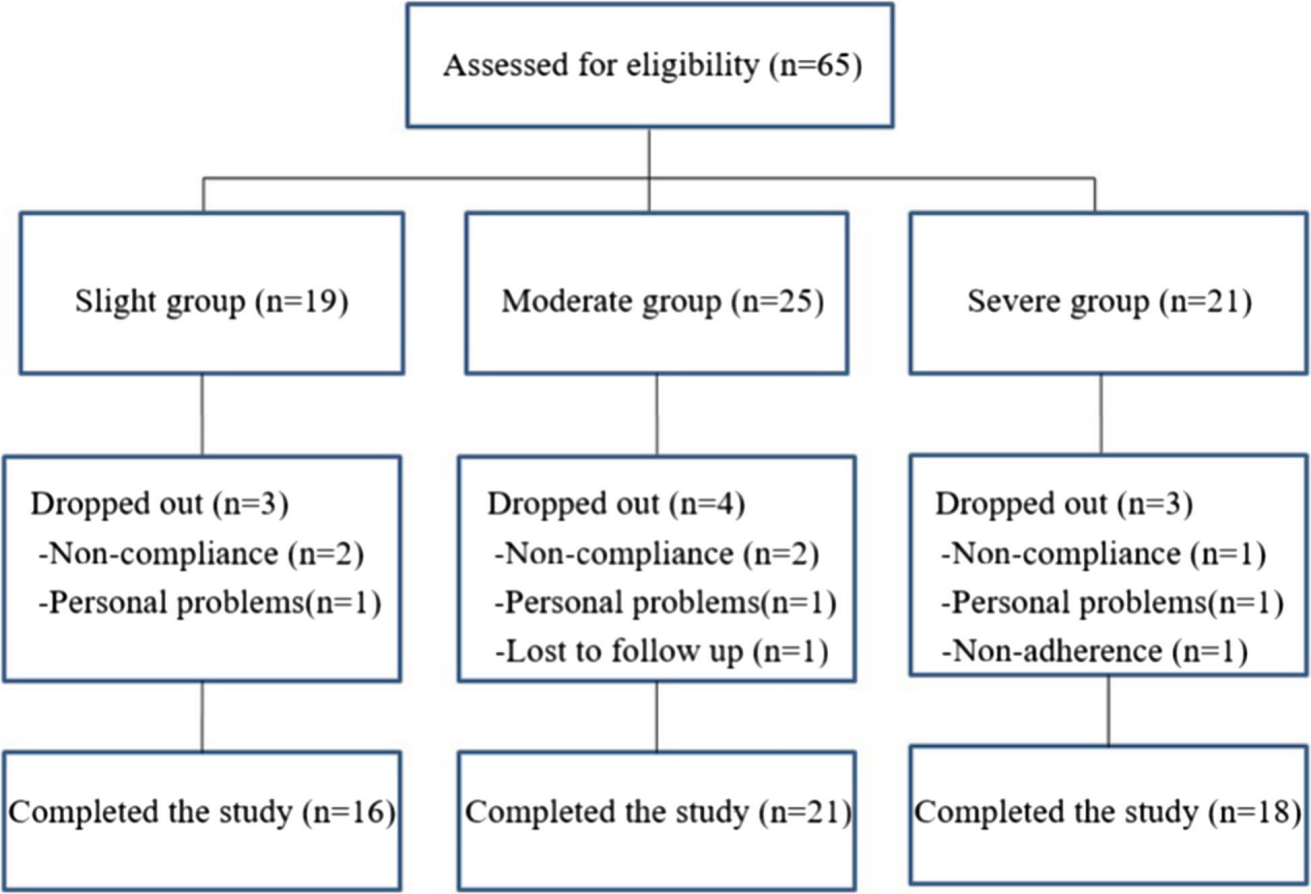
The flow diagram of included and excluded patients

We screened 65 subjects, including slight group (*n* = 19), Moderate group (*n* = 25), and Severe group (*n* = 21). However, ten subjects dropped out due to non-compliance, personal problems, lack of follow up, and non-adherence to drug therapy. Ultimately, 21 men and 34 women (mean age 38.27 ± 11.47 years) were enrolled (Fig. 1).

There were no significant differences among the three groups regard to age or duration of illness on HAMD. According to the patient’s condition, the medication prescription of the patients was recorded. Furthermore, there is no difference in the educational experience and smoking status. In addition, the baseline characteristics of all groups are summarized in Table 1.

**Table 1:** Baseline characteristics of the participants

	Slight	Moderate	Severe	*P*-value
Participants	16	21	18	
Age (years)	35.16 ± 11.81	39.33 ± 10.95	39.83 ± 11.85	0.43
Gender (M/F)	7/9	8/13	6/12	0.82
Education				0.96
Below 9 years, n (%)	1 (6.25)	2 (9.5)	1 (5.5)	
Up to 9 years, n (%)	5 (31.3)	7 (33.3)	8 (44.4)	
10–12 years, n (%)	3 (18.8)	2 (9.5)	2 (11.1)	
13–17 years, n (%)	7 (43.8)	10 (47.6)	7 (38.9)	
Smoking, n (%)	2 (12.5)	3 (14.3)	3 (16.7)	0.94
First-episode, n (%)	10 (62.5)	13 (61.9)	9 (50)	0.69
Baseline HAMD score	21.94 ± 4.60	22.09 ± 5.04	22.94 ± 8.16	0.88
Baseline SCL 90 score	200.31 ± 53.69	205.38 ± 70.08	219.56 ± 67.57	0.72
Baseline ISI score	10.31 ± 2.94	16.81 ± 1.50	23.56 ± 1.62	
Current treatment				0.78
Sertraline	5	7	5	
Fluvoxamine	6	4	4	
Escitalopram	2	5	6	
Fluoxetine	3	5	3	

*HAMD* Hamilton Depression Scale, *ISI* Insomnia Severity Index, *SCL-90* Symptom Checklist-90

### Outcomes

**Fig. 2:**
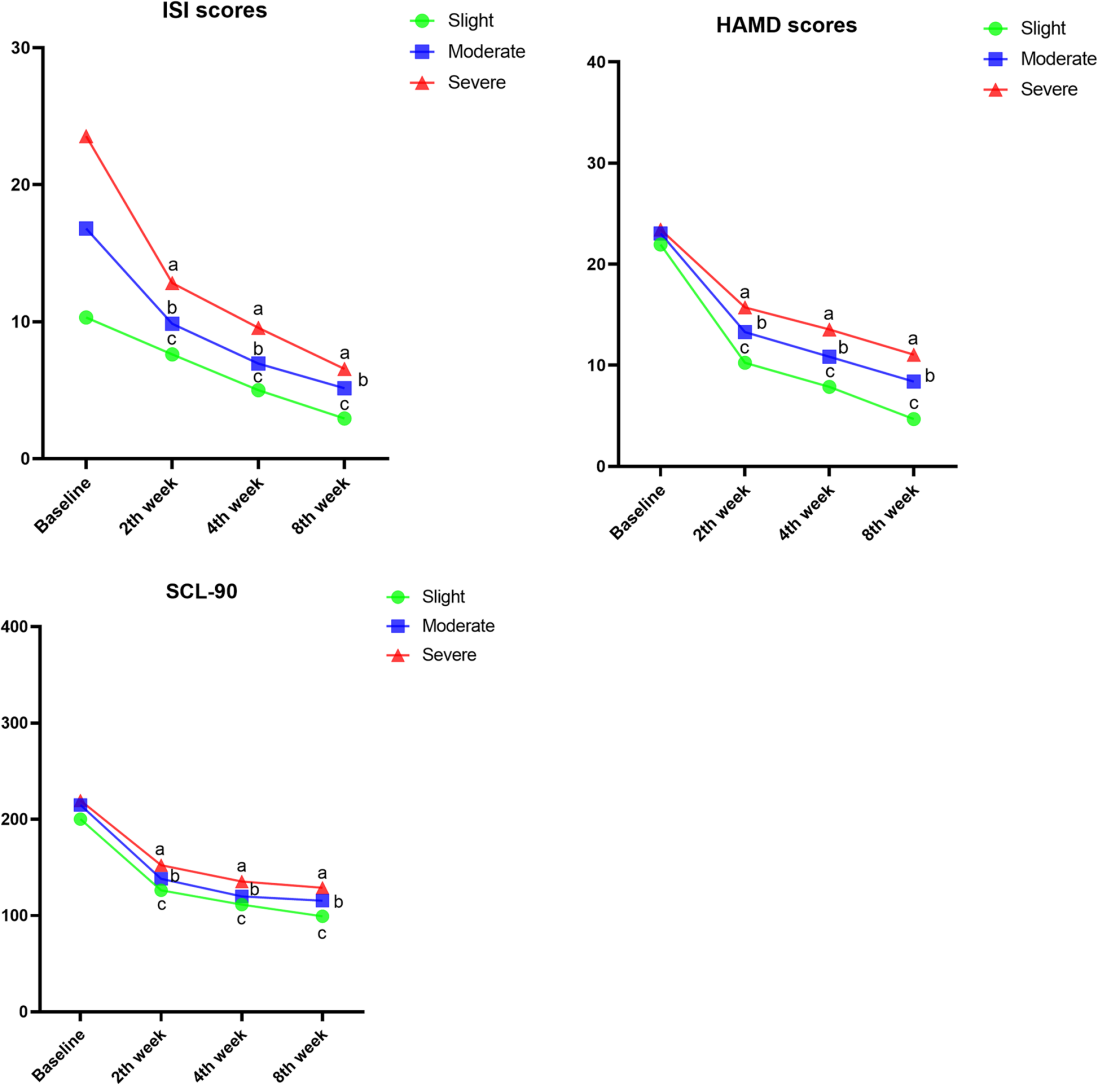
Scores on the ISI, HAMD and SCL-90 in time of 2nd, 4th, and 8th-week follow-up. ^a^ indicate *P* < 0.05 versus the baseline of the severe group; ^b^ indicate *P* < 0.05 versus the baseline of the moderate group; ^c^ indicate *P* < 0.05 versus the baseline of the slight group. HAMD, Hamilton Depression Scale; ISI, Insomnia Severity Index; SCL-90, Symptom Checklist-90

The effects of insomnia on the prognosis of patients with MDD are shown in Fig. 1. The sleep quality of MDD patients improved beginning in the 2nd week and gradually improved with the increase of prognosis time. The ISI scores of the moderate and severe group decreased significantly (*P* < 0.05) in the 2nd week compared to the baseline. The scores of all groups improved significantly at 8 weeks (Fig. 2).

The HAMD scores in all groups improved significantly in the second week. In the 4th and 8th weeks, the change from baseline in HAMD score of the slight group was higher than that of the severe group (Table 2). In the 8th week, significant differences were observed between the moderate and severe groups in comparison to the slight group by the response HAMD (χ^2^ = 6.13; *P*<0.05) and remission HAMD (χ^2^ = 13.32; *P*<0.05). As shown in Table 3, we observed the change from baseline in SCL-90 score of the slight group (100.93 ± 48.85) was higher than that of the severe group (90.66 ± 58.54) in the 8th week. Furthermore, the trends of ISI, HAMD, and SCL-90 are similar.

**Table 2:** Change from baseline in HAMD score. Remission: final HAMD score ≤ 7. Response: 50% reduction in the pre-post-intervention

	Slight	Moderate	Severe	χ^2^	*P*-value
2nd week follow-up	10.25 ± 4.31	7.88 ± 2.55	4.69 ± 2.77		
Change from baseline in HAMD score	11.69 ± 4.46	9.76 ± 4.83	7.72 ± 6.22		
Response HAMD, n	8	5	3	5.02	*P* = 0.081
Remission HAMD, n	5	1	1	6.98	*P*<0.05
4th week follow-up	13.29 ± 4.11	10.85 ± 4.30	8.38 ± 3.71		
Change from baseline in HAMD score	14.07 ± 3.99	12.2 ± 4.89	9.88 ± 6.65		
Response HAMD, n	13	11	10	3.65	*P* = 0.16
Remission HAMD, n	6	5	5	0.85	*P* = 0.66
8th week follow-up	15.72 ± 5.02	13.56 ± 6.29	11.06 ± 6.99		
Change from baseline in HAMD score	17.25 ± 4.01	14.67 ± 4.72	12.38 ± 6.97		
Response HAMD, n	15	14	12	6.13	*P*<0.05
Remission HAMD, n	14	7	6	13.32	*P*<0.05

**Table 3:** Change from baseline in SCL-90 score

	Slight	Moderate	Severe
2nd week follow-up	126.4 ± 31.08	138.2 ± 44.23	152.3 ± 54.03
Change from baseline in SCL-90 score	73.91 ± 46.69	67.18 ± 61.38	67.26 ± 61.92
4th week follow-up	111.4 ± 24.45	120.1 ± 30.63	135.6 ± 46.02
Change from baseline in SCL-90 score	88.91 ± 46.56	85.28 ± 60.85	83.96 ± 59.78
8th week follow-up	99.38 ± 11.88	115.5 ± 21.48	128.9 ± 35.27
Change from baseline in SCL-90 score	100.93 ± 48.85	89.88 ± 62.19	90.66 ± 58.54

## Discussion

This study demonstrates the effect of insomnia on patients with MDD. According to ISI, HAMD, and SCL-90 scores, the sleep quality of MDD patients improved beginning in the 2nd week and gradually improved with the increase of prognosis time. In addition, there is a recovery consistency between insomnia and MDD. It is a study to demonstrate that there is a clear correlation between insomnia and MDD.

Insomnia is a syndrome defined as difficulty in falling asleep, maintaining sleep, or non-restorative sleep [14]. There is a bidirectional relationship between insomnia and mood symptoms, poor sleep may precede the episode of MDD, and depressed mood disrupt standard sleep patterns [15]. In addition, people with insomnia are three times more likely to suffer from MDD than those without insomnia [16]. Insomnia is a condition caused by other disorders that are completely independent and can lead to coexisting disorders. Mental disorders, circadian rhythm disorders, or other sleep disorders may be the disorders [17, 18]. In this study, 55 MDD patients with insomnia were enrolled. According to the ISI scores, they were divided into three groups. The results showed no significant differences among the three groups regarding HAMD scores. In the prognosis of MDD, insomnia may be of great significance in preventing the recovery of the disorder.

In routine practice, clinicians have a wide choice of individual drugs, and need good evidence to make the best choice for each patient as Selective serotonin reuptake inhibitors (SSRIs), sertraline, fluvoxamine, escitalopram, and fluoxetine have been indicated to be effective for MDD [19]. In addition, evidence has suggested that sertraline and fluoxetine have similar insomnia efficacy [20]. From a clinical trial, fluvoxamine appears to be beneficial for treating insomnia in MDD [21]. In addition, previous studies have shown that escitalopram could improve subjective sleep experience [22, 23]. In terms of drug selection, we selected these four drugs according to the best choice for each patient. These SSRIs have beneficial effects on sleep disorders. It indicates that insomnia coexists with MDD.

Insomnia is the main symptom of several common sleep disorders, but it often coexists with mental illnesses. In addition, insomnia can affect the trajectory of MDD and increase the severity and duration of this disorder [14]. The poor subjective sleep quality before the treatment of MDD may indicate a reduced treatment response. It has been reported that MDD patients with interpersonal therapy have higher sleep quality scores than that with no remission and significantly improved mood [24]. Furthermore, poor sleep quality is related to poor response to depressed therapy [25]. In addition, it has been reported that the severity of insomnia is one of the clinical features predicting suicide within 1 year, and this connection has been reported in adolescence [26]. Adolescents with insomnia have the most severe MDD, and those with insomnia have more severe MDD than those without sleep disturbance [27]. Sleep disturbances are also associated with an increased risk of suicide in adolescents [28, 29]. Therefore, we can recognize the depth impact of insomnia on MDD. Our results showed that the sleep quality improved beginning in the 2nd week and gradually improved with the increase of time. The HAMD scores in all groups improved significantly in the second week. Moreover, the slight group performed best. It can see that insomnia is closely related to the prognosis of MDD. In the recovery process of depression, better sleep quality is beneficial to the recovery of MDD.

It has been indicated that insomnia is closely connected to depressive symptoms, resulting in poor prognosis conditions. Insomnia-related symptoms are essential and modifiable risk factors for achieving and maintaining MDD remission. Based on the above results, clinicians need to evaluate the sleep symptoms of MDD patients carefully. Insomnia commonly coexists with MDD rather than following MDD. We believe that insomnia and MDD need require specific treatment. The evidence confirms that we should pay more attention to insomnia while treating MDD.

## Conclusions

This study was inspired to assess insomnia as a comorbid disorder for patients with MDD, which may bring poor treatment consequences. This study suggests that severe sleep disorders may have poor implications on the prognosis of MDD.

## Data Availability

The datasets generated and/or analyzed during the current study are not publicly available due to confidentiality but are available from the corresponding author on reasonable request.
